# Tumor budding as an indicator of prognosis in locally advanced rectal cancer after neoadjuvant chemoradiotherapy: a systematic review and meta-analysis

**DOI:** 10.3389/fonc.2025.1429319

**Published:** 2025-04-09

**Authors:** Azita Rafiee, Parto Nasri, Afshin Moradi, Paridokht Karimian

**Affiliations:** ^1^ Department of Pathology, Iranian Medical and Pathology Laboratory, Zahedan, Iran; ^2^ Department of Pathology, Isfahan University of Medical Sciences, Isfahan, Iran; ^3^ Cancer Research Center, Shahid Beheshti University of Medical Sciences, Tehran, Iran; ^4^ Department of Pathology, School of Medicine, Guilan University of Medical Sciences, Rasht, Iran

**Keywords:** rectal neoplasm, neoadjuvant therapy, tumor budding, prognosis, colon cancer

## Abstract

**Introduction:**

Tumor budding (TB) is recognized as a complementary prognostic factor for colorectal cancer. However, data on its impact on the survival of patients undergoing neoadjuvant chemoradiotherapy (nCRT) remain limited. This study aims to investigate the role of TB in disease-free survival (DFS) and overall survival (OS) among patients with locally advanced rectal cancer receiving nCRT.

**Methods:**

In this systematic review and meta-analysis, an exhaustive search of the PubMed, Scopus, Web of Science (WOS), Embase, and Cochrane databases was conducted, ultimately leading to the extraction of eight studies in the qualitative assessment and meta-analysis.

**Results:**

All the included studies were of high quality. The total sample size comprised 1,941 individuals. Although eight studies were included, nine datasets were extracted, as some studies reported multiple outcome measurements. TB positivity was statistically associated with decreased overall survival of 3.24 (95% confidence interval [CI]: 1.71–6.16) and disease-free survival of 2.54 (95% CI: 1.56–4.15) in patients with locally advanced rectal cancer undergoing nCRT.

**Discussion:**

Based on the findings of this study, TB negativity was statistically and directly associated with better OS and DFS in patients with locally advanced rectal cancer undergoing nCRT.

## Introduction

Malignancies have become a major public health challenge, representing the second leading cause of mortality worldwide (1). Among men, lung, prostate, and colorectal cancer (CRC) contribute significantly to cancer-related deaths, while in women, breast, lung, and CRC account for more than half of all malignancies. In 2020, CRC was ranked as the third most prevalent malignancy and the second leading cause of cancer-related mortality. Additionally, a gradual increase in CRC-associated deaths was observed between 2005 and 2020 across both age groups—those under and over 50 years old (2).

The histopathological analysis of CRC specimens indicates that adenocarcinoma is the most prevalent type, accounting for 95% of all CRC cases, originating from cellular proliferation and dysplasia of polyps (3). The most widely used and robust classification system applied for assessing the extent and clinical outcome of CRC is TNM staging, which plays a crucial role in determining the appropriate treatment approach, including local excision, neoadjuvant therapy, and major surgical resection (4–6).

The preferred approach for treating locally advanced rectal cancers classified as T3–T4 and/or N+, M0 according to TNM staging is total mesorectal excision (TME) combined with neoadjuvant chemoradiotherapy (nCRT) (4–6). Neoadjuvant chemoradiotherapy is generally defined as either long-course chemoradiotherapy, long-course chemoradiotherapy following primary chemotherapy, or short-course radiotherapy (7, 8).

Nevertheless, the variability in outcomes among CRC patients within the same TNM stage, even after undergoing complete radical surgery, has led to the hypotheses that additional factors may play a crucial role in assessing treatment response beyond tumor staging and the chosen therapeutic approach. Tumor budding (TB) is one of the pathological characteristics suspected to contribute to this variability. Furthermore, since TNM staging is based on pathological examination, it can only be applied to resected specimens, limiting its usage in planning and adjusting neoadjuvant therapy (9, 10).

TB is a morphological marker of epithelial–mesenchymal transition (EMT) (11) and is defined as a single cancer cell or a cluster of fewer than five cells located at the invasive front of the tumor (peritumoral budding) or within the tumor mass (intratumoral budding). These cells tend to lose adhesion, making the tumor more invasive (12). A review of the literature indicates that TB is associated with adverse tumoral characteristics, including higher tumor grade, higher TNM stage, lymphovascular invasion, lymph node involvement, distant metastasis, and overall shorter survival (13–17). The prognostic significance of TB is so pronounced that it has been suggested to be a stronger predictor of survival than ypT and ypN staging (18).

Nevertheless, a comprehensive investigation into the value and utility of TB as a predisposing factor for adverse outcomes in locally advanced rectal cancer patients undergoing neoadjuvant therapy is lacking. This meta-analysis aims to address this gap.

## Materials and methods

### Research strategy

We aimed to conduct a systematic review and meta-analysis following the Preferred Reporting Items for Systematic Reviews and Meta-Analyses (PRISMA) guidelines (19). To ensure a comprehensive search, we utilized the PubMed, Scopus, Web of Science (WOS), Embase, and Cochrane databases up to 12 August 2023. The databases were searched using keywords derived from Medical Subject Heading (MeSH) terms extracted from MeSH on Demand. These keywords were then entered into the databases according to their respective protocols, followed by a reference search. Our initial search yielded 480 articles.

The searched keywords included: (“tumour budding”) OR (“tumor budding”) OR (“high-grade tumor budding”) OR (“low-grade tumor budding”) OR (“budding”) OR (“tumor-cell dissociation”) and (colorectal neoplasms) OR (colorectal neoplasm) OR (colorectal tumors) OR (colorectal tumor) OR (colorectal cancer) OR (colorectal cancers) OR (colorectal carcinoma) OR (colorectal carcinomas) OR (rectal carcinomas) OR (rectal carcinoma) OR (rectal neoplasms) OR (rectal neoplasm) OR (rectal tumors) OR (rectal tumor) OR (rectal cancers) OR (rectal cancer) OR (“CRC”) and (neoadjuvant therapy) OR (neoadjuvant radiotherapy) OR (neoadjuvant radiation treatment) OR (neoadjuvant radiation therapy) OR (neoadjuvant radiation) OR (neoadjuvant systemic therapy) OR (neoadjuvant systemic treatment) OR (neoadjuvant chemotherapy) OR (neoadjuvant chemotherapy treatment) OR (neoadjuvant chemoradiotherapy) OR (neoadjuvant chemoradiation therapy) OR (neoadjuvant chemoradiation treatment) OR (neoadjuvant chemoradiation).

### Inclusion criteria

The studies included in this meta-analysis met the following criteria: (1) written in English, (2) assessed relapse-free survival (RFS) or disease-free survival (DFS) or overall survival (OS) in patients with locally advanced rectal adenocarcinoma, (3) involved patients who received any neoadjuvant chemoradiotherapy treatments, and (4) provided histopathological reports of TB.

### Exclusion criteria

Studies were excluded from this meta-analysis if they lacked sufficient data for analysis, reported patients with tumors other than rectal adenocarcinoma, had inaccessible full texts, were classified as low-quality studies, were review articles, were written in languages other than English, or were individual case reports.

### Study selection

The authors (A.R., P.N., and P.K.) compiled and reviewed the topics of the manuscripts. The topics and names of the first authors were then checked. Next, EndNote software was used to eliminate duplicate manuscripts. Following this, the three authors independently reviewed the abstracts and selected relevant articles for inclusion. In cases where there was disagreement regarding the inclusion of a particular manuscript, another author (A.M.) made the final decision. Finally, the full text of the selected manuscripts was assessed for eligibility, evaluated for quality and risk of bias, and included in the meta-analysis.

### Population, intervention, comparison, and outcome components

#### Population

This study focuses on patients diagnosed with locally advanced rectal cancer who have undergone neoadjuvant therapy.

#### Intervention

Tumor budding assessment was performed to evaluate its prognostic significance in patients receiving neoadjuvant therapy.

#### Comparison

Patients were compared based on different levels of tumor budding to determine its impact on clinical outcomes.

#### Outcome

The study examines overall survival, disease-free survival, and relapse-free survival in patients with locally advanced rectal cancer in relation to tumor budding status.

### Data extraction

The authors independently extracted data from the included papers, including the first author, year of publication, studied population, study type, applied protocol for nCRT, and TB reporting system.

### Statistical analysis

To demonstrate effect size as a standardized mean difference between the tumor-budding-positive and tumor-budding-negative individuals receiving neoadjuvant chemoradiotherapy, overall survival or disease-free survival/relapse-free survival was used as the effect size measure in all studies and represented in a forest plot. The meta-analysis was conducted using declared generic, precomputed effect sizes based on mean for two-group comparisons of continuous or binary outcomes. All effect sizes, including relative risk, odds ratio, beta regression, and correlation, were converted to standardized mean difference (SMD). Also, the random-effects restricted maximum-likelihood (REML) model was applied. Substantial heterogeneity was indicated by an *I* ^2^ value > 50% and a Cochran’s square test, *H* ^2^, with a corresponding *p*-value of < 0.05. Galbraith plots were used to evaluate the sources of heterogeneity among studies. Subgroup analysis was performed using RFS/DFS or OS. Since DFS and RFS have similar definitions, they were analyzed as a subgroup (10, 20). A sensitivity analysis test was performed to assess the robustness of the associations. Publication bias was evaluated using a funnel plot, Egger’s test, and Begg’s tests. A nonparametric trim-and-fill analysis was conducted to estimate the number of missing studies. All data analyses were performed using Stata software version 17.

## Results

### Study selection

**Figure 1:**
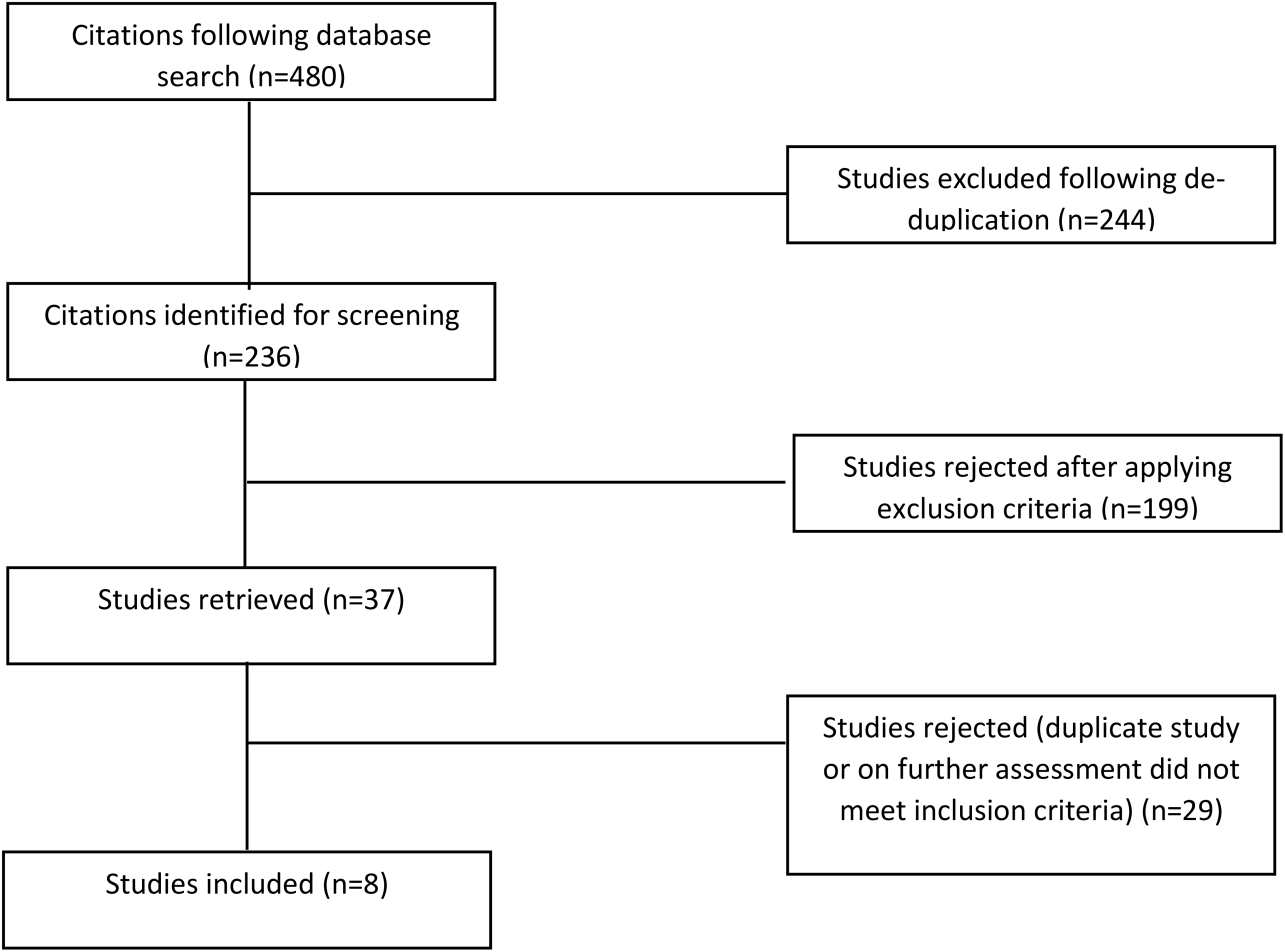
Flowchart illustrating a summary of literature search results.

The literature search strategy identified 480 records, of which 236 remained after duplicate removal. Following an initial screening of titles and abstracts, 37 full-text articles were retained and assessed. Ultimately, eight studies were included in the qualitative assessment and meta-analysis, as shown in the PRISMA flow diagram (**Figure 1**) and **Table 1**.

**Table 1:** Characteristics of the included studies.

First author (year)	Sirin et al. (2019) (21)	Huebner et al. (2012) (23)	Jager et al. (2018) (10)	Farchoukh et al. (2021) (24)
**Type of study**	Prospective	Retrospective	Retrospective	Retrospective
**Country**	Turkey	USA	Austria	USA
**Study period**	2000–2010	1996–2006	2003–2012	2010–2019
**Number of patients received CRT/sex (male)**	117 CRT	237 CRT/160 male patients	128 CRT/87 male patients	117 CRT/69 male patients
**Age (years)**	–	60.0 ± 12.5	64 (34–84)	–
**Interval to surgery (week) after CRT**	8	6–8	5.1 (2.7–9.3)	–
**Tumor stage**	T3–T4/N0 or Tany N+ (stages I–III)	I, II, III	T3–T4 and/or N+ (stages 0–III)	0–III
**Neoadjuvant therapy regimen**	1.8 Gy/day, 5 days/week, for a total of 25 fractions over 5 weeks, for a total of 4,500 + 5-fluorouracil (FU) at 225 mg/m^2^/day for 5 days/week within 5 weeks	Irradiation and 5-FU	45–50 GY over 5–6 weeks with concurrent 5-FU, capecitabine, and oxalipalatine	(1) Radiotherapy (50.4 Gy) with concomitant 5-FU (2) Systemic 5-FU, leucovorin, and oxaliplatin (FOLFOX) followed by radiotherapy with concomitant 5-FU
**TB assay method**	TB was assessed using the H&E staining method by scanning for the hot spot area, followed by counting at × 400 magnification.	TB was assessed at the tumor edge using the H&E staining method at × 200 magnification.	TB was evaluated using the H&E staining method by scanning at low magnification (× 4 up to × 10), and the average number of TB in 10 HPF (×40) was calculated.	TB was assessed using the H&E staining method, following a modification of the Rogers et al. method and the hot spot method of ITBCC.
**TB sorting method in the study**	- TB-1: none (0) and mild (1–5 buds) - TB-2: moderate (6–10 buds) and severe (> 10 buds)	- Negative: ≤ 9 buds/field - Positive: ≥ 10 buds/field	- None: no TB - Mild: ≤ 1 TB - Moderate: 1 < TB < 5 - Severe: ≥ 5 TB - BD-0: none or mild TB - BD-1: moderate or severe TB	- Absent: < 2 TB/0.785 mm^2^ area - Present: ≥ 2 TB/0.785 mm^2^ area
**Follow-up period**	40.12 ± 27.5 months	Median follow-up: 3.5 years (25th and 75th percentiles: 2.1 and 4.8 years)	7 years (2.9–146.7 months)	29 months (3–106 months)
**Hazard ratio of OS**	4.87 (2.1–11.28)	–	–	–
**Hazard ratio of DFS/RFS**	–	2.45 (1.14–5.3)	3.44 (1.23–9.63)	3.35 (1.25–8.99)

First author (year)	Rogers et al. (2014) (25)	Shin et al. (2021) (22)	Sannier et al. (2014) (26)	Trotsyuk et al. (2019) (18)	Trotsyuk et al. (2019) (18)
**Type of study**	Prospective	Retrospective	Retrospective	Retrospective	Retrospective
**Country**	Ireland	Korea	France	Germany	Germany
**Study period**	2003–2010	2007–2014	2005–2010	2002–2011	2002–2011
**Number of patients received CRT/sex (male)**	89 CRT/57 male patients	939 CRT/633 male patients	113 CRT/67 male patients	103 CRT/73 male patients	99 CRT/71 male patients
**Age (years)**	62 (30–84)	–	59.2 ± 11.1 (28–84)	–	–
**Interval to surgery (week) after CRT**	6–8	6 and 8	6–9	4–6	4–6
**Tumor stage**	–	I, II, III	T3–T4 and/or N+	T2–T4, NO-N+	T2–T4, NO-N+
**Neoadjuvant therapy regimen**	45–50.4 Gy in 25–28 fractions of 1.8 Gy over 6 weeks with concomitant 5-FU	4,500–5,400 cGy over 5–6 weeks with concomitant 5-FU	45–50 Gy over 5–6 weeks with concomitant 5-FU	50.4 Gy delivered in 5 weekly fractions of 1.8 Gy using 18 MeV photons, with concurrent infusion of 225 mg 5-FU per day	50.4 Gy administered in 5 weekly fractions of 1.8 Gy using 18 MeV photons, with concurrent infusion of 225 mg 5-FU per day
**TB assay method**	TB was assessed using the H&E staining method at × 40 magnification, with positive cases confirmed at × 100 magnification according to the Giger et al. method	TB was counted at the invasive front of the tumor using the H&E staining method	TB was assessed at the invasive tumor margin and within the tumor body using the H&E staining method	TB was assessed in the hot spot area using the H&E staining method and counted with a × 20 objective	TB was assessed in the hot spot area using the IHC staining method and counted with a × 20 objective
**TB sorting method in the study**	- Positive vs. negative - Any TB confirmed at × 100 magnification was considered positive	- Negative: TB < 5 buds - Positive: TB ≥ 5 buds	- Presence vs. absence of TB - The presence of only 1 TB in any field is considered significant	- Negative (BD-0): TB ≤ 4 buds/0.785 mm^2^ - Positive (BD-1): TB ≥ 5 buds/0.785mm^2^	- Negative (BD-0): TB ≤ 4 buds/0.785 mm^2^ - Positive (BD-1): TB ≥ 5 buds/0.785 mm^2^
**Follow-up period**	49 months (7–117 months)	5 years	35.2 months (1–72 months)	54.7 ± 35.5 months	54.7 ± 35.5 months
**Hazard ratio of OS**	–	2.102 (1.11–9.97)	–	2.72 (1.15–6.44)	5.19 (1.62–16.61)
**Hazard ratio of DFS/RFS**	–	1.66 (1.1–2.5)	–	2.34 (1.14–4.79)	4.59 (1.79–11.72)

In the meta-analysis, separate forest plots were generated for RFS/DFS and OS outcomes to assess the role of TB in the prognosis of rectal cancer.

The quality and risk of bias assessment of the included studies indicated that all studies were of good quality (**Table 2**) and had a low risk of bias (**Table 3**).

**Table 2:** The included studies’ quality.

Number	Author	Study design	1	2	3	4	5	6	7	8	9	10	11	12	13	14	Quality
**1**	Şirin (2019) (21)	Cohort	✔	✔	✔	✔	✔	✔	✔	✔	✔	✔	✔	✔	✔	✔	Good
**2**	Shin (2021) (22)	Cohort	✔	✔	✔	✔	✔	✔	✔	✔	✔	✔	✔	–	✔	✔	Good
**3**	Sannier (2014) (26)	Cohort	✔	✔	✔	✔	✔	✔	✔	✔	✔	✔	✔	–	✔	✔	Good
**4**	Rogers (2014) (25)	Cohort	✔	✔	✔	✔	✔	✔	✔	✔	✔	✔	✔	✔	✔	✔	Good
**5**	Jäger (2018) (10)	Cohort	✔	✔	✔	✔	✔	✔	✔	✔	✔	✔	✔	✔	✔	✔	Good
**6**	Huebner (2012) (23)	Cohort	✔	✔	✔	✔	✔	✔	✔	✔	✔	✔	✔	–	✔	✔	Good
**7**	Farchoukh (2021) (24)	Cohort	✔	✔	✔	✔	✔	✔	✔	✔	✔	✔	✔	✔	✔	✔	Good
**8**	Trotsyuk (2019) (18)	Cohort	✔	✔	✔	✔	✔	✔	✔	✔	✔	✔	✔	✔	✔	✔	Good

✔, this symbol is used when the cited study meets the 14 criteria of the quality assessment.

**Table 3:** Assessing bias in the included studies using the QUIPS tool.

Number	Author	Study design	Rating of bias domains
**1**	Şirin (2019) (21)	Cohort	Low risk
**2**	Shin (2021) (22)	Cohort	Low risk
**3**	Sannier (2014) (26)	Cohort	Low risk
**4**	Rogers (2014) (25)	Cohort	Low risk
**5**	Jäger (2018) (10)	Cohort	Low risk
**6**	Huebner (2012) (23)	Cohort	Low risk
**7**	Farchoukh (2021) (24)	Cohort	Low risk
**8**	Trotsyuk (2019) (18)	Cohort	Low risk

The total sample size comprised 1,941 individuals. Although there were eight studies, we extracted data from nine studies, as one study assessed outcomes using two different staining methods (18) (**Table 1**).

The main characteristics and data reported in the included articles are summarized in **Table 1**. All the studies were cohort-based and conducted on human samples. The recruited studies were from Turkey (21), South Korea (22), the USA (23, 24), Ireland (25), Austria (10), France (26), and Germany (18) (**Table 1**).

Tumor budding was evaluated in patients with locally advanced rectal cancer undergoing neoadjuvant chemoradiotherapy using histopathological specimens stained with hematoxylin and eosin or immunohistochemistry. Studies defined tumor budding according to different classification systems, with some reporting TB as positive or negative, while others classified it based on a cut-off or continuous scale. TB positivity was determined using various thresholds, including TB ≥ 1 (21, 25, 26), ≥ 2 (10, 24), ≥ 5 (18, 22), and ≥ 10 (23). The final assessment of the studies included 3-year (26) or 5-year (10, 18, 21–25) DFS/RFS and OS.

### Study quality

The National Heart, Lung, and Blood Institute (NIH) Study Quality Assessment Tool was used to evaluate the study quality, categorizing all included studies as good quality, as shown in **Table 2**. Moreover, the Quality In Prognosis Studies (QUIPS) tool was applied to assess validity and risk of bias, considering six bias domains: study participation, study attrition, prognostic factor measurement, outcome measurement, study confounding, statistical analysis, and reporting. Each domain was rated as low, moderate, or high risk of bias based on prompting items and considerations (27). Following the evaluation, all included studies were categorized as having a low risk of bias (**Table 3**).

### Outcomes

#### Relapse-free survival/disease-free survival

**Figure 2:**
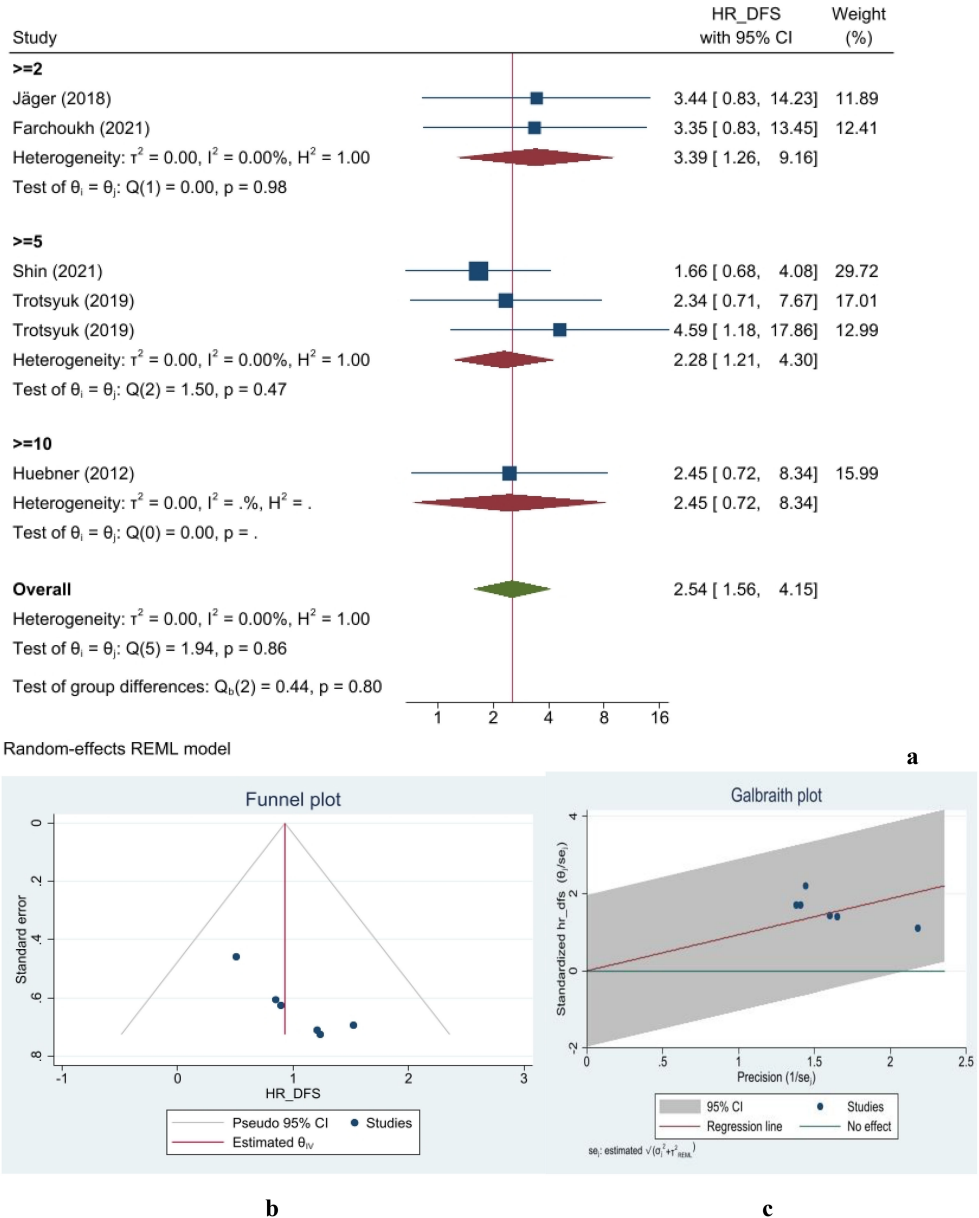
Forest **(a)**, funnel **(b)**, and Galbraith **(c)** plots for disease-free survival/relapse-free survival.

Data from six studies on DFS, including 1,623 patients, were incorporated into this meta-analysis. Based on the definition of TB positivity, the overall hazard ratio for DFS in TB-negative patients was 2.54 times higher than in TB-positive individuals, as estimated using a random-effects model (95% confidence interval [CI]: 1.56–4.15) (**Figure 2a**). Moreover, no publication bias was observed in any of the analyses. The gathered data were generally homogeneous, with an *I* ^2^ value of 0%. The assessment of group differences showed no significant statistical differences (*p*-value = 0.86) (**Figure 2b**). The figure illustrates the distribution of studies in the assessment of DFS, indicating that none fell outside the 95% confidence interval limits. The small-study effect was evaluated using Begg’s and Egger’s tests. In the Egger’s test, a beta value of 3.15 was calculated with a corresponding *p*-value of 0.19. The Begg’s test yielded a Kendall’s τ of 11, with a two-tailed *p*-value of 0.06. Finally, the nonparametric trim-and-fill analysis indicated no missing studies affecting specificity measurements. Galbraith plot of RFS/DFS is shown in **Figure 2c**.

#### Overall survival

**Figure 3:**
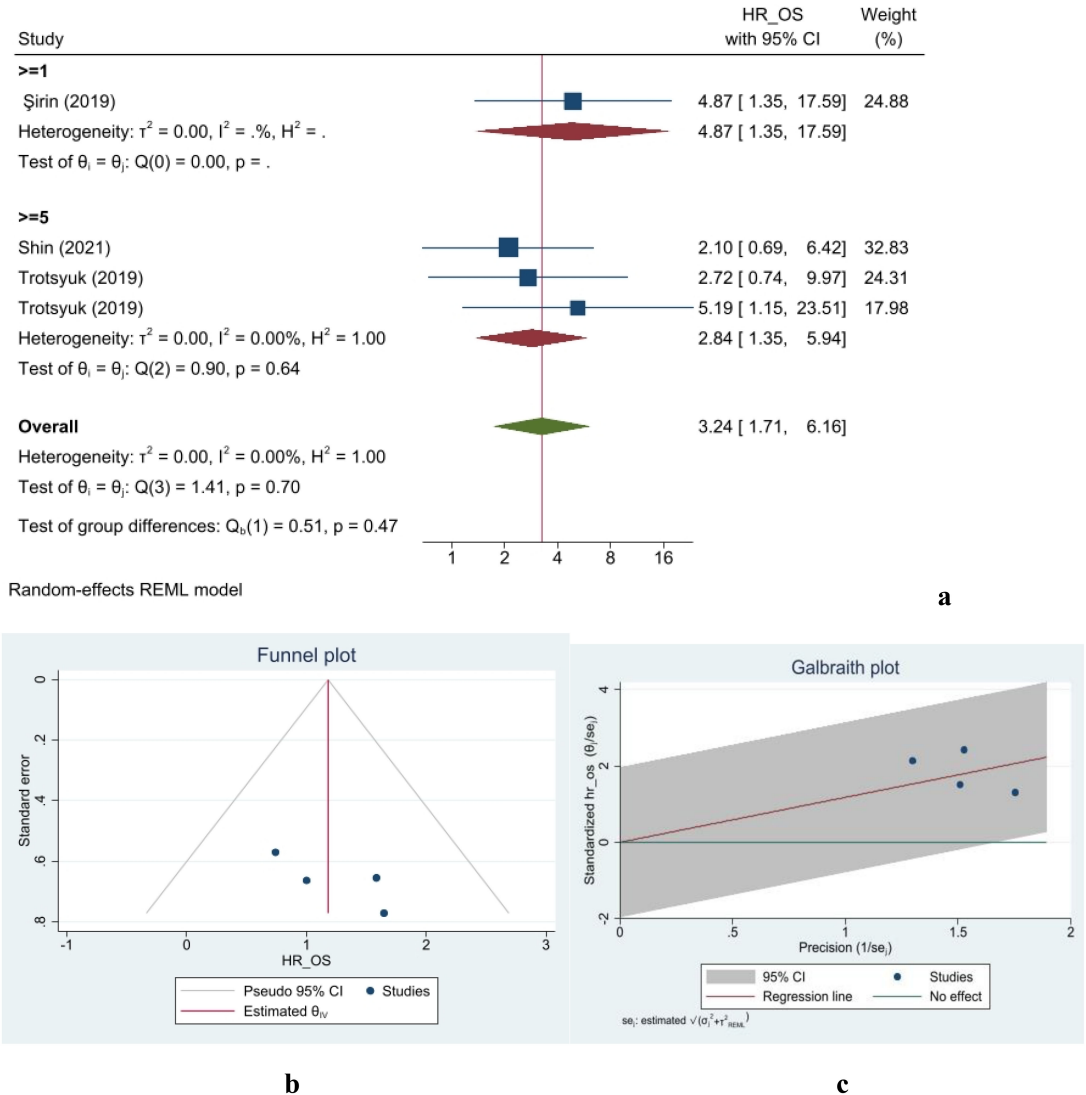
Forest **(a)**, funnel **(b)**, and Galbraith **(c)** plots for overall survival.

Data from four studies on OS, including 1,257 patients, were incorporated into this meta-analysis. The overall measured hazard ratio indicated a 3.24-fold increase in overall survival for TB-negative patients compared with TB-positive cases (95% CI: 1.71–6.16) (**Figure 3a**). Moreover, no publication bias was observed in any of the analyses. The data showed no heterogeneity, with an *I* ^2^ of 0%. The assessment of group differences revealed no statistical significant differences (*p*-value = 0.70). **Figure 3b** illustrates the dispersion of studies in the assessment of OS, showing that none fell outside the 95% confidence interval limits. The small-study effect was evaluated using Begg’s and Egger’s tests. The Egger’s test yielded a beta value of 4.54 with a corresponding *p*-value of 0.33. In the Begg’s test, Kendall’s τ was 2, with a two-tailed *p*-value of 0.73. Finally, a nonparametric trim-and-fill analysis indicated no missing studies affecting specificity measurements. Galbraith plot of OS is shown in **Figure 3c**.

## Discussion

Despite significant progress in colorectal cancer treatment, patient responses to therapeutic approaches vary, suggesting the influence of additional factors. Recent studies have highlighted TB as a potential determinant colorectal cancer invasion and response to nCRT, given its notable prognostic value in lymph node involvement, distant metastasis, local recurrence, and 5-year cancer-associated mortality in patients undergoing primary surgical resection without nCRT (17). TB is often an under-recognized pathological factor in colorectal cancer, but its importance is underscored by studies showing that high-grade TB correlates with upregulation of negative regulatory immune checkpoints (PD-L1, TIM-3) and chemokine receptors (CXCR2, CXCR4) (28), which are associated with poor prognosis in patients with colorectal cancer liver metastasis undergoing neoadjuvant chemotherapy (29). Given its significance, TB has been recommended for inclusion in future CRC reporting guidelines/protocols and the next TNM staging system as a prognostic factor for colorectal cancers (12).

Despite a few studies assessing the prognostic value of TB in patients with locally advanced rectal cancer undergoing nCRT, to the best of our knowledge, the current investigation is the first systematic review and meta-analysis assessing evaluating this parameter in these cases. Our study revealed that TB negativity, regardless of its variable scoring systems in the studies, was associated with a 2.54- and 3.24-fold increase in DFS and OS, respectively.

In more detailed information, Huebner and colleagues were the first to evaluate the prognostic value of TB in assessing response to nCRT. While they did not specify the intensity of radiation used in their chemoradiotherapy protocol, they reported administering radiotherapy in combination with 5-fluorouracil (5-FU) as the chemotherapy regimen for patients with stages I–III rectal cancer undergoing nCRT. Their assessment of TB’s role in RFS demonstrated a 2.46-fold increase in RFS among TB-negative patients (23).

Research on TB continued with two studies in 2014, conducted by Sannier et al. in France (26) and Rogers et al. in Ireland (25). Rogers reported a 5-year DFS of 33% for TB-positive subjects vs. 77% for TB-negative subjects. They further indicated that TB predicted a poor pathological response to nCRT, as it was associated with adverse conditions such as higher ypT stage, lymph node involvement, lymphovascular invasion, and poorly differentiated tumors (25). Similarly, Sannier identified TB as a prognostic factor for failure to respond to nCRT in patients with types III–IV rectal cancer who had positive node involvement (26).

Similarly, Jäger and colleagues reported significantly better outcomes for TB-negative patients, with a 5-year RFS of 90% and a distant recurrence rate of 2%, compared to 71% and 12% for TB-positive individuals. Furthermore, TB positivity was identified as a negative predictive factor for RFS (HR: 3.44). However, their results did not show a significant association between BD-1 and OS. Notably, they classified TB as negative and mild TB (BD-0) vs. moderate and severe TB (BD-1) (10).

The latter study by Şirin et al. categorized TB into four groups: none (0), mild (1–5 buds), moderate (6–10 buds), and severe (> 10 buds). Their findings on the association between TB and OS were consistent with previous studies, showing a 4.28-fold decrease in OS. However, TB was not identified as an independent prognostic factor for DFS. Notably, they did not provide HR details in their analysis (21).

One of the most notable confirmatory studies in this area was conducted by Trotsyuk et al., who evaluated this hypothesis using two methods of staining: hematoxylin and eosin and immunohistochemistry. While both methods produced consistent results, immunohistochemistry assessments higher OS and DFS for TB-negative individuals. Additionally, they emphasized that TB was a superior predictor of overall survival compared to traditional parameters such as ypT and ypN status (18).

The latest studies in this area have indicated that TB positivity is associated with a poor response to nCRT, with a 5-year DFS of 87% in patients without TB compared to 39% in those with TB (24). Shin et al. (22) supported these findings, emphasizing an earlier theory that TB status not only determines the response to nCRT but also independently predicts disease outcome, OS, and DFS, regardless of nCRT treatment.

Despite the value of the findings in the current study, several notable challenges should not be overlooked. Accordingly, the limitations of this meta-analysis, which may also introduce potential sources of bias, should be considered. Primarily, the number of studies assessing the prognostic value of TB for DFS, OS, and response to nCRT in locally advanced rectal cancer is limited. Secondly, the TB scoring system varies considerably between studies. Although the prognostic significance of TB remains largely independent of the scoring system used, establishing a single international standard for TB assessment is necessary for consistency in reporting (12). Most studies recommended the hot spot method (a single field with the highest number of TB), while others used multiple field methods (e.g., 5 HPF and 10 HPF) (13, 15, 16). However, efforts have been made to establish a standardized definition, as outlined in the International Tumor Budding Consensus Conference (ITBCC) published in 2016 (12). The ITBCC recommended the hot spot method for counting TB, in which the invasive front is scanned at 10 medium power fields (× 10 objective)to identify the hot spot (the area containing the highest number of TB) in the initial step. Next, TB should be counted in a single × 20 objective field within the hot spot area, and the TB count is then calculated in an area measuring 0.785 mm^2^ using a normalization factor (12). To minimize the risk of bias in TB counting, the ITBCC recommends a continuous scale, which is more precise than the cut-off method (30). Furthermore, the ITBCC suggests a three-tier scoring system to categorize TB as low (BD-1: 0–4), intermediated (BD-2: 5–9, and severe (BD-3: ≥ 10) budding (12). The third point of discussion concerns the different staining methods used to assess tumor budding. Although only one of the studies used immunohistochemistry to assess tumor budding, variations in staining methods might influence the outcomes. However, it has been suggested that the prognostic power of H&E and IHC staining methods in evaluating TB does not differ (13, 15–17, 31). Furthermore, the ITBCC has noted that H&E is comparably favored over the methods (12).

Our study also provides new insights into the association between the presence of TB and a reduced response to nCRT in locally advanced rectal adenocarcinoma.

## Conclusion

Based on the findings of the current study, TB negativity was statistically and directly associated with better OS and DFS in patients with locally advanced rectal cancer undergoing nCRT. If further studies confirm the role of TB in reducing the response to nCRT and decreasing OS and DFS in these patients, TB may be serve as an indication for modifying and individualizing nCRT regimens for locally advanced rectal adenocarcinoma.

## Data Availability

The raw data supporting the conclusions of this article will be made available by the authors, without undue reservation.
